# Open-label extension study of leniolisib in activated PI3Kδ syndrome: Long-term efficacy and safety

**DOI:** 10.70962/jhi.20260040

**Published:** 2026-10-14

**Authors:** V. Koneti Rao, Anna Šedivá, Virgil A.S.H. Dalm, Alessandro Plebani, Catharina Schuetz, Anna Shcherbina, Antonio Trizzino, Yulia Zharankova, Alanvin Orpia, Elaine Kulm, Julia Körholz, Vassilios Lougaris, Yulia Rodina, Jason Bradt, Anurag Relan, Gulbu Uzel

**Affiliations:** 1 Laboratory of Clinical Immunology and Microbiology, National Institute of Allergy and Infectious Disease, National Institutes of Health, Bethesda, MD, USA; 2 Department of Immunology, 2nd Faculty of Medicine, Charles University and Motol and Homolka University Hospital, Prague, Czech Republic; 3 Department of Internal Medicine, Division of Allergy & Clinical Immunology and Department of Immunology, Erasmus MC University Medical Center, Rotterdam, Netherlands; 4 Pediatrics Clinic, Department of Clinical and Experimental Sciences, University of Brescia, ASST Spedali Civili of Brescia, Brescia, Italy; 5 Department of Pediatrics, Medizinische Fakultät Carl Gustav Carus, Technische Universität Dresden, Dresden, Germany; 6 Department of Immunology, Dmitry Rogachev National Medical Research Center of Pediatric Hematology, Oncology and Immunology, Moscow, Russia; 7 Department of Pediatric Hematology and Oncology, ARNAS Civico Di Cristina Benfratelli Hospital, Palermo, Italy; 8 Belarusian Research Center for Pediatric Oncology, Hematology and Immunology, Minsk, Belarus; 9 National Institute of Allergy and Infectious Diseases, National Institutes of Health, Bethesda, MD, USA; 10 Clinical Research Directorate, Frederick National Laboratory for Cancer Research, Bethesda, MD, USA; 11 Department of Pediatrics, Carl Gustav Carus Faculty of Medicine, Dresden University of Technology, Dresden, Germany; 12 Pharming Healthcare Inc., Warren, NJ, USA; 13 Laboratory of Clinical Immunology and Microbiology, National Institute of Allergy and Infectious Diseases, National Institutes of Health, Bethesda, MD, USA

## Abstract

Activated phosphoinositide 3-kinase delta syndrome (APDS) is an inborn error of immunity characterized by immune deficiency and dysregulation. Leniolisib is a selective phosphoinositide 3-kinase delta inhibitor approved to treat APDS in patients ≥12 years old. We report final results from a single-arm, open-label extension study in 37 patients with APDS (NCT02859727). Median leniolisib exposure was 4.3 years (range, 1.2–7.0 years). Adverse events (AE) were reported in 34 patients; 87% were grade 1. Serious AEs, reported in 10 patients, were deemed unrelated to leniolisib. Two patients discontinued treatment due to AEs: cardiac arrest and Hodgkin’s lymphoma. There were significant reductions in annual infection (P = 0.0005) and respiratory infection (P = 0.0017) rates per year of treatment. Immunoglobulin replacement therapy (IRT) use significantly decreased per year of treatment (P = 0.0095). Clinically meaningful improvements in general health scores from the 36-Item Short Form Survey were observed. Overall, long-term use of leniolisib was generally well tolerated, and infections and IRT use reduced over time.

## Teaser

Leniolisib, a PI3Kδ inhibitor, improves the pathophysiology of APDS. This publication demonstrates the safety and efficacy of leniolisib in patients with APDS ≥12 years of age receiving up to 7 years of treatment, illustrating a positive tolerability profile and sustained disease burden reduction.

## Graphical Abstract

**Figure:**
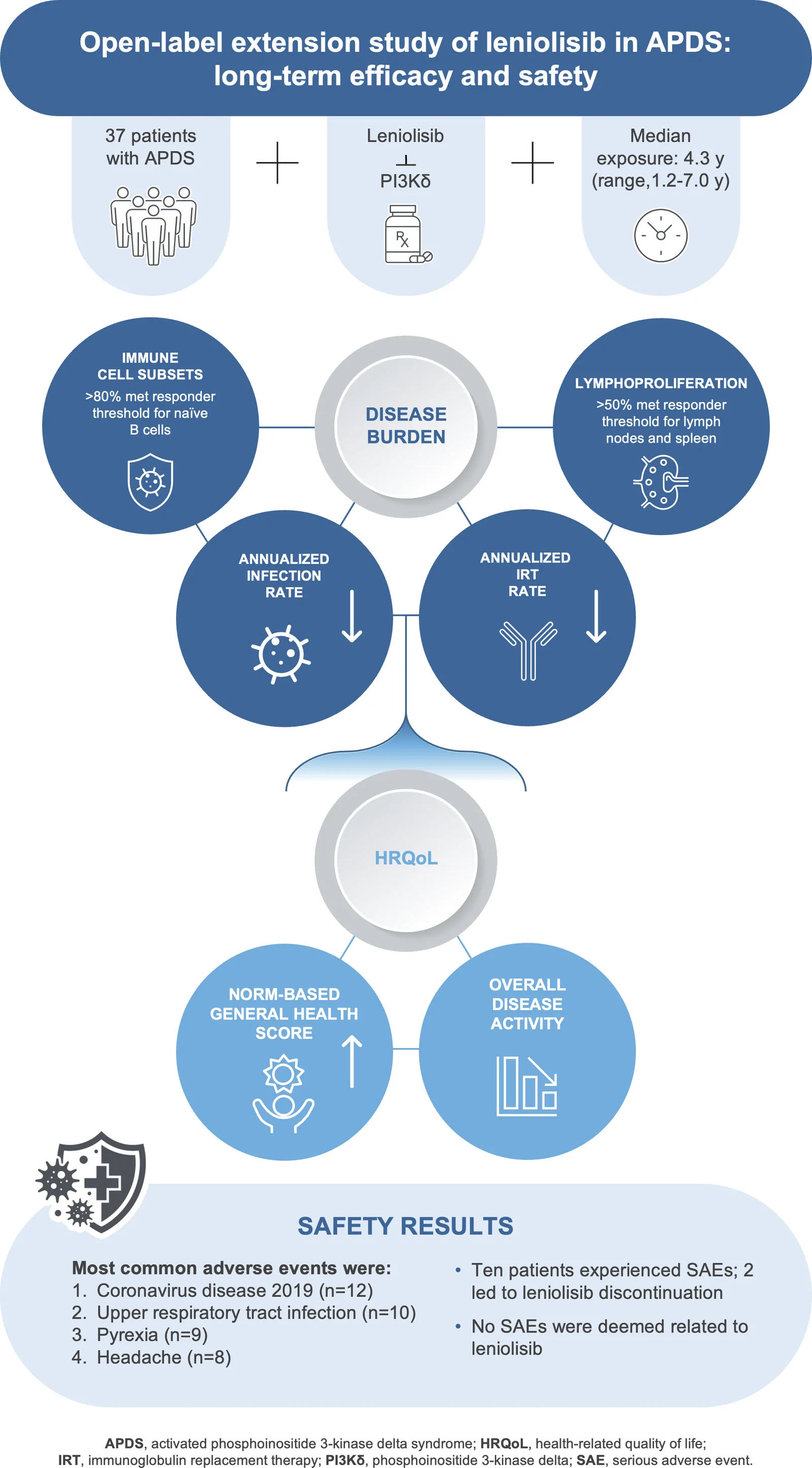

## Introduction

Activated phosphoinositide 3-kinase delta (PI3Kδ) syndrome (APDS) is an inborn error of immunity (IEI) driven by hyperactive PI3Kδ signaling, caused by pathogenic heterozygous gain-of-function variants in *PIK3CD* or loss-of-function variants in *PIK3R1*, which encode the kinase (p110δ) and regulatory (p85α) subunits of the PI3Kδ heterodimer, respectively (1, 2, 3). PI3Kδ hyperactivation alters lymphocyte development and function, causing immune deficiency and dysregulation (4, 5, 6). Pathognomonic B and T cell subsets contribute to clinical manifestations, including recurrent sinopulmonary infections, herpesvirus viremia, chronic lymphoproliferation, and autoimmunity, which can lead to end-organ damage including bronchiectasis, liver disease, and hearing loss (7, 8, 9). Additionally, patients have an increased risk of developing lymphoma, in part due to uncontrolled antigen- or EBV-driven PI3Kδ hyperactivity in B cells and impaired T cell–mediated tumor surveillance (8, 10, 11). APDS is a progressive disease that is not only associated with a cumulative disease burden and reduction in quality of life (QoL) but also early mortality (12, 13, 14).

Traditional management strategies for APDS are empirical and include immunoglobulin replacement therapy (IRT), prophylactic antimicrobials, immunomodulatory therapies such as mammalian target of rapamycin (mTOR) inhibitors, surgeries, and hematopoietic stem cell transplantation (4, 5, 7). PI3Kδ can be selectively inhibited with leniolisib; as of January 2026, leniolisib is approved in the US, UK, Israel, and Australia to treat patients ≥12 years of age with APDS (15, 16, 17, 18). Results from a dose-finding trial (DFT) and phase 3, placebo-controlled trial (NCT02435173) showed normalization of the PI3Kδ signaling pathway and pathognomonic B and T cell subsets, as well as a reduction in lymphoproliferation at 12 wk (19, 20). Long-term safety and efficacy of leniolisib was examined in a single-arm, open-label extension (OLE) study (NCT02859727). In previous interim analyses of this study, patients with APDS received leniolisib for up to 5 and 6 years (presented at the International Primary Immunodeficiencies Congress 2025), respectively (21). Results revealed that long-term use of leniolisib was generally well tolerated and patients experienced durable improvements in disease markers, including lymphocytes and lymphoproliferation. Here, we report final safety and efficacy data from the OLE in patients with APDS that received leniolisib for up to 7 years.

## Results

### Baseline demographics and leniolisib exposure

**Figure 1.**
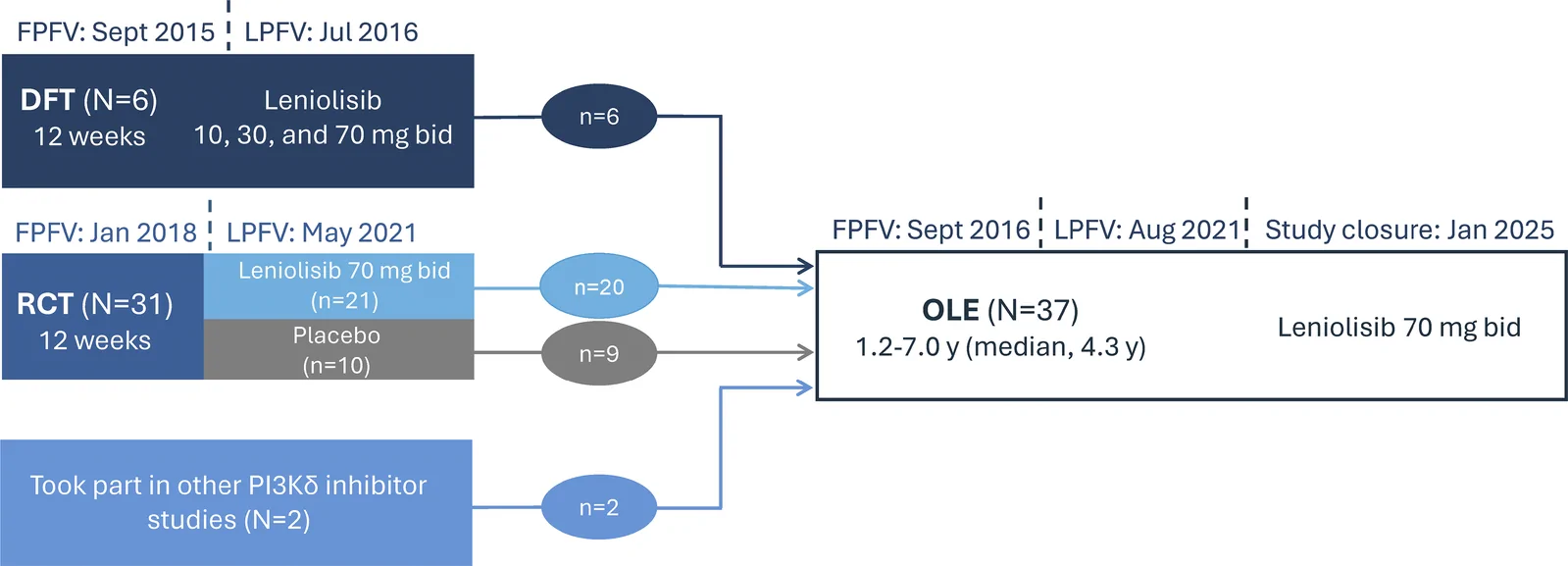
**Study design and patient enrollment.** Six patients received leniolisib during the initial DFT. These six patients did not take part in the randomized placebo-controlled trial, which included 21 patients in the leniolisib arm and 10 in the placebo arm. All six patients from the DFT enrolled in the OLE. 20 patients from the leniolisib arm and 9 from the placebo arm rolled into the OLE. Additionally, two new patients who previously received PI3Kδ inhibitors in other clinical studies enrolled in the OLE. bid, twice daily; FPFV, first patient first visit; LPFV, last patient first visit; RCT, randomized placebo-controlled trial.

In total, 37 participants enrolled in the OLE study (Table S1). The study included 21 male and 16 female patients with a genetically confirmed variant in either *PIK3CD* (*n* = 30) or *PIK3R1* (*n* = 7) and clinical symptoms compatible with APDS. Two patients previously received PI3Kδ inhibitors in other clinical studies, while all others participated in either the DFT or randomized controlled trial (RCT; Fig. 1). 26 patients (70%) were previously treated with leniolisib.

Medical history reported at extension day (ED) 1 included infections (*n* = 32), lymphoproliferation (*n* = 25), cytopenia (*n* = 20), chronic gastrointestinal disease (*n* = 19), chronic pulmonary disease (*n* = 21), and fatigue (*n* = 12). Baseline medications of interest were examined; 27 patients (73.0%) received IRT, 22 (59.5%) antibiotics, and 21 (56.8%) glucocorticoids. Sirolimus was previously administered in eight patients (21.6%).

**Figure 2.**
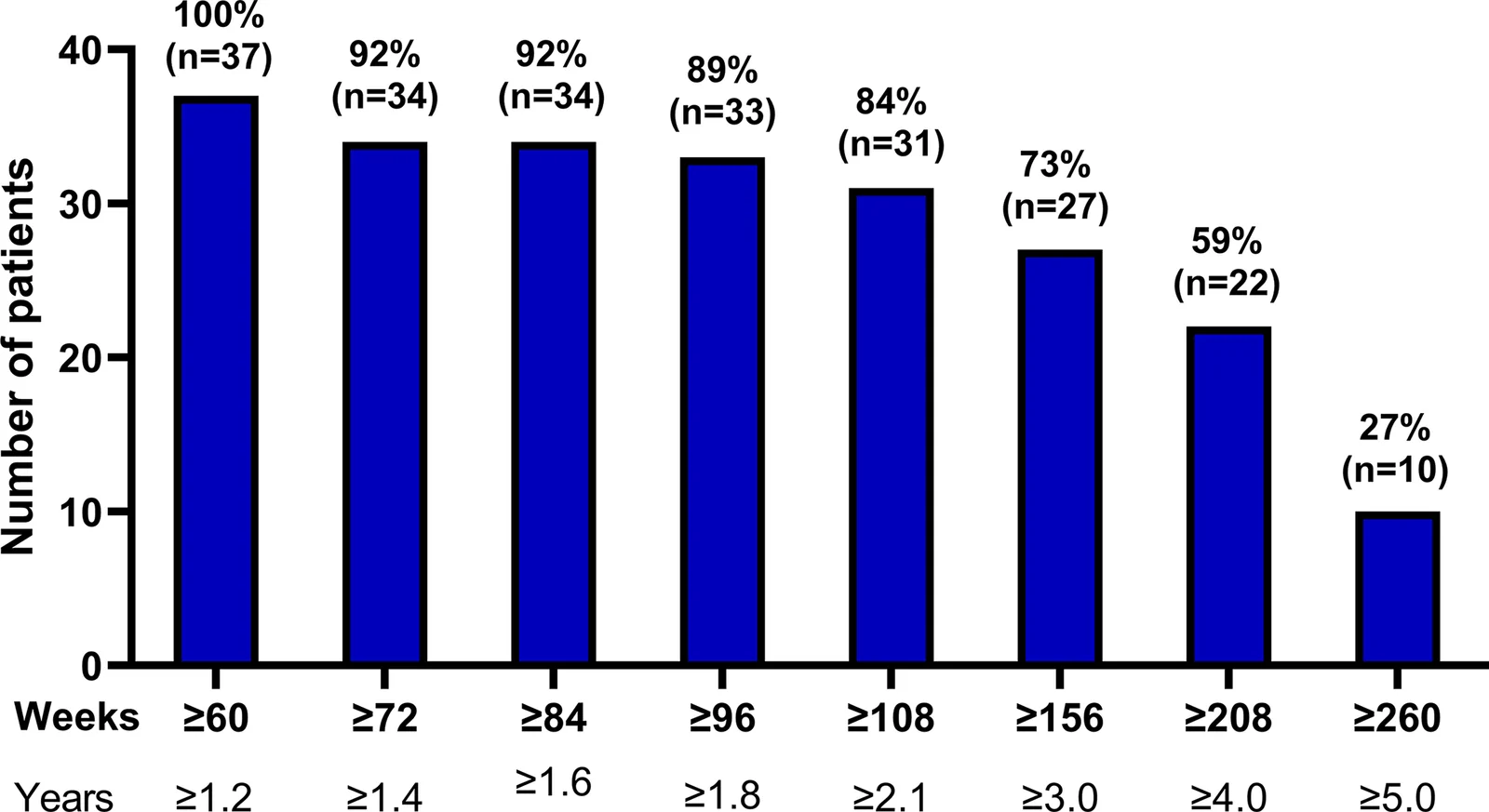
Minimum duration of exposure to leniolisib.

By study closure (January 2025), median leniolisib exposure was 4.3 years (range, 1.2–7.0 years); 22 patients (59%) had ≥4 years of exposure (Fig. 2). Three patients (8.1%) completed the study as planned (Table 1). Six participants (16.2%) discontinued the study for the following reasons: death (*n* = 1), adverse event (AE; *n* = 1), lost to follow-up (*n* = 2), and subject or guardian decision (*n* = 2). Regarding the latter two patients, one discontinued prior to pregnancy while the other did so due to improvements in health. Of the remaining 28 patients, 3 discontinued the study due to physician decision and 25 due to sponsor study termination; all transitioned to either commercial use of leniolisib in the United States or under expanded access or compassionate use programs. Overall, 31 patients (83.8%) continued leniolisib treatment following the completion of the OLE.

**Table 1.** Patient disposition (*N* = 37)

Status	Patients, *n* (%)
**Enrolled until official study termination**	3 (8.1)
**Discontinued before official study termination** ^a^	34 (91.9)
**Continuing leniolisib outside study** ^b^	31 (83.8)
**Discontinued leniolisib during study**	6 (16.2)
AE	1 (2.7)
Death	1 (2.7)
Lost to follow-up	2 (5.4)
Subject/guardian decision	2 (5.4)

a Patients in this category did not necessarily discontinue leniolisib.

b Includes patients on commercial drug and through an early-access program.

### Safety

**Figure 3.**
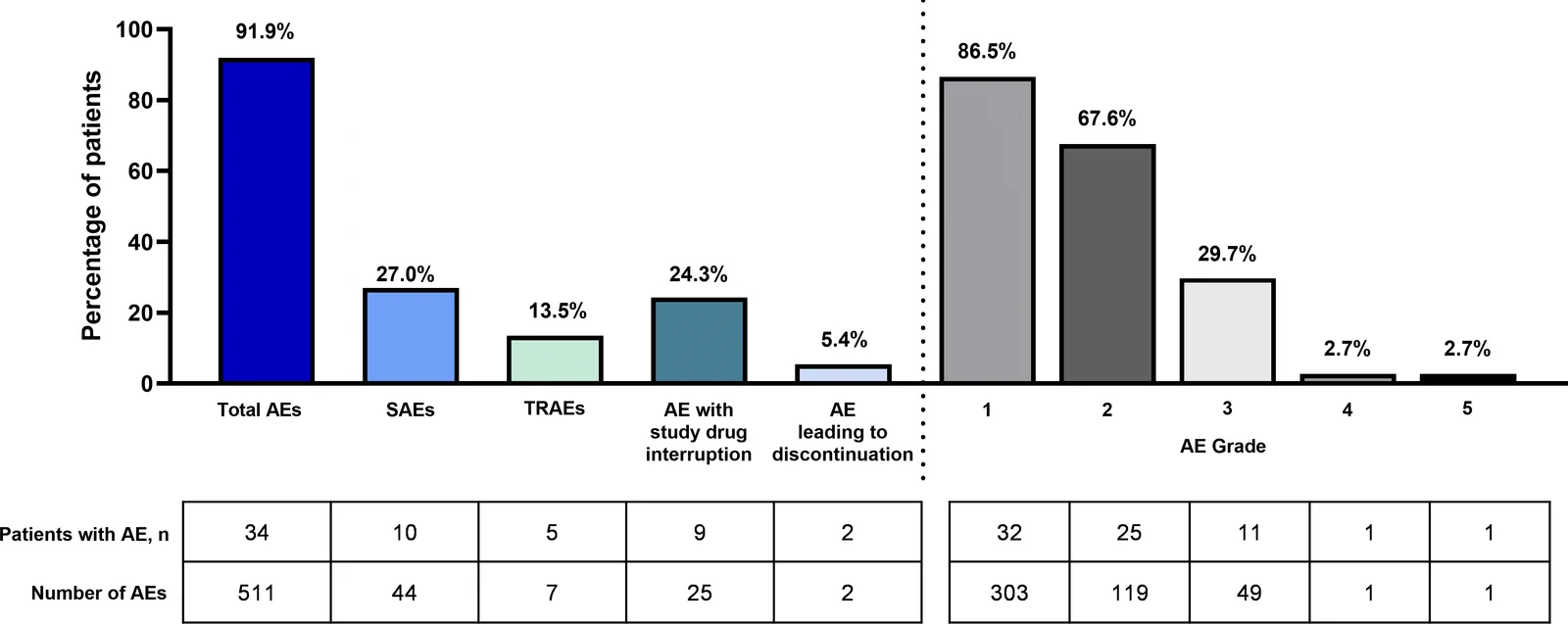
**AEs and AE grades.** SAE, serious AE.

AEs were reported in 34 patients (92%) and were largely grade 1 (86.5%; Fig. 3). The most common AEs were coronavirus disease 2019 (*n* = 12), upper respiratory tract infection (*n* = 10), pyrexia (*n* = 9), and headache (*n* = 8; Table S2). Regarding AEs of interest, 1 of 17 patients with bronchiectasis experienced an exacerbation during the OLE that occurred within the first 2 wk of switching from placebo to leniolisib. Additionally, lymphadenopathy was reported in two patients and deemed unrelated to leniolisib. Mild lymphadenopathy was reported in a 23-year-old patient during year 4 of treatment and resolved. Serious lymphadenopathy (grade 3) was reported in a 15-year-old patient during year 3, and as a result, treatment was interrupted for 4 days. After 2 mo, lymphadenopathy resolved. Overall, serious AEs (Table S3) were reported in 10 patients (27%), with 6 of these patients reporting multiple serious AEs. None were deemed related to leniolisib. Two serious AEs led to treatment discontinuation, including one patient with significant baseline comorbidities who experienced cardiac arrest (grade 5), resulting in death at ED 879, as previously reported (21). Another patient who received placebo in the RCT was diagnosed with stage IV Hodgkin’s lymphoma (grade 3) at ED 750 and discontinued treatment. This 20-year-old patient had a history of EBV infections and missed several doses of leniolisib during the OLE study, with an approximate 2-mo gap in treatment during the first year. The only grade 4 serious AE reported was suicidal ideation at ED 1147 in a 15-year-old patient who previously received leniolisib in the RCT. The patient was hospitalized for 7 days and, upon discharge, the event resolved with no recurrence by the end of the OLE study. Another notable serious AE was suspected transitional cell carcinoma (grade 3) in a 55-year-old patient at ED 559, at which point leniolisib was interrupted and resumed upon event resolution 144 days later. None of the AEs described were deemed related to leniolisib.

Treatment-related AEs (TRAEs) were reported in five patients, with one of these patients experiencing multiple TRAEs. Events included weight increase (*n* = 3), arthralgia (*n* = 1), hyperglycemia (*n* = 1), and neutrophil count decrease (*n* = 1). Notably, no new TRAEs were reported since the first interim analysis (21).

### Infections

**Figure 4.**
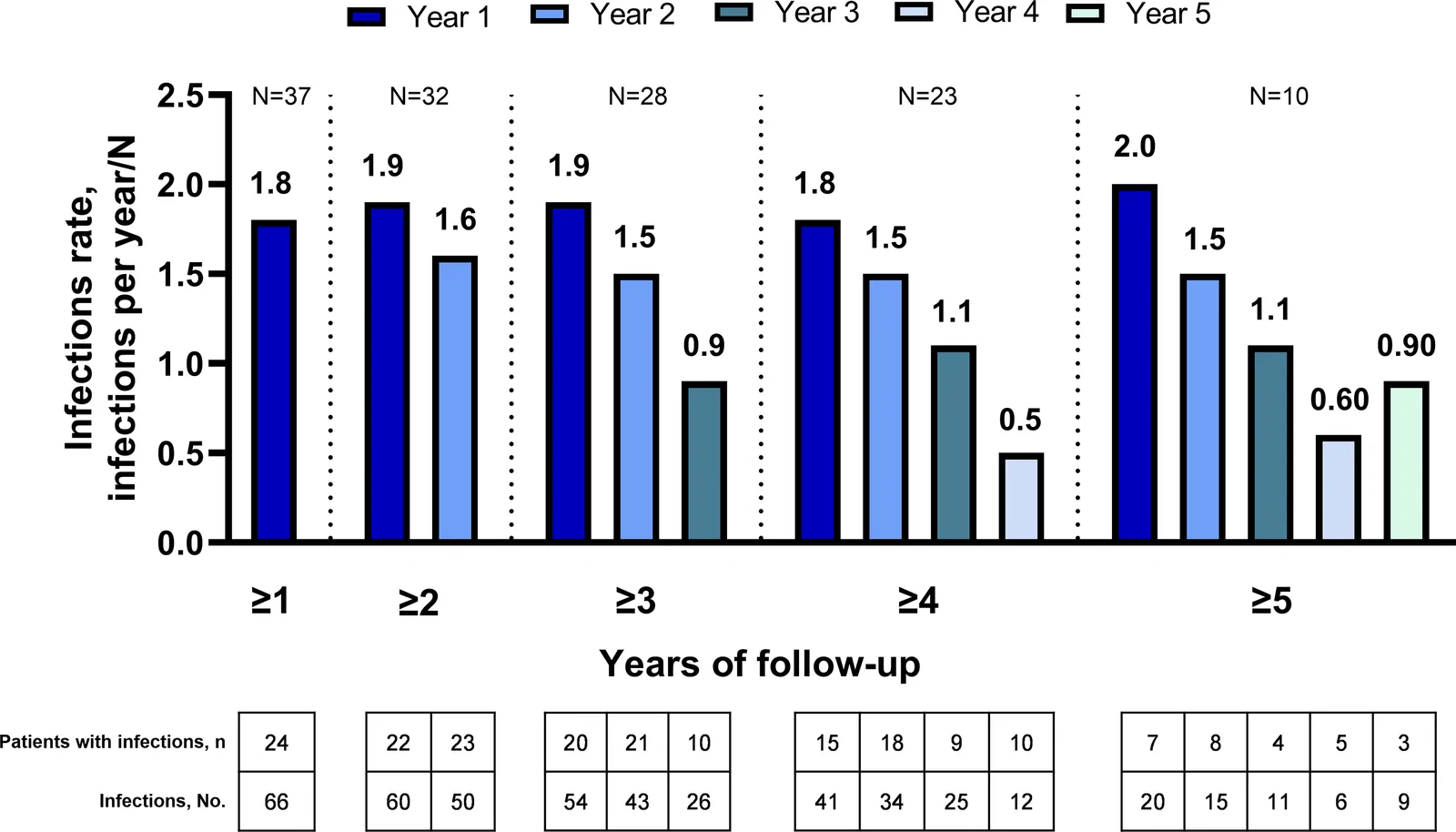
**Infection rate per year of treatment with leniolisib.** *N*, number of participants in the follow-up category.

At the end of the OLE study, a total of 181 infections were reported in 31 patients (84%) including 120 respiratory infections reported in 28 patients (76%). Per additional year of leniolisib treatment, there was a 25% reduction in infection rates (slope = −0.282; P = 0.0005; Fig. 4). Similarly, there was also a 25% reduction in respiratory infection rates with each additional year of leniolisib treatment (slope = −0.284; P = 0.0017).

Assessment of EBV and CMV from ED 1 to the end of treatment revealed that the highest viral loads were ∼50,119 and 6,310 copies/ml, respectively. Prior medical history of EBV viremia was reported in the patient with the highest viral load of EBV, and chronic CMV infections were reported in the patient with the highest viral load of CMV prior to treatment with leniolisib. The latter patient started valganciclovir for chronic CMV infections prior to entry into the trials and, as of the final data cut of the OLE, continues to receive this treatment.

### IRT and antibiotic use

**Figure 5.**
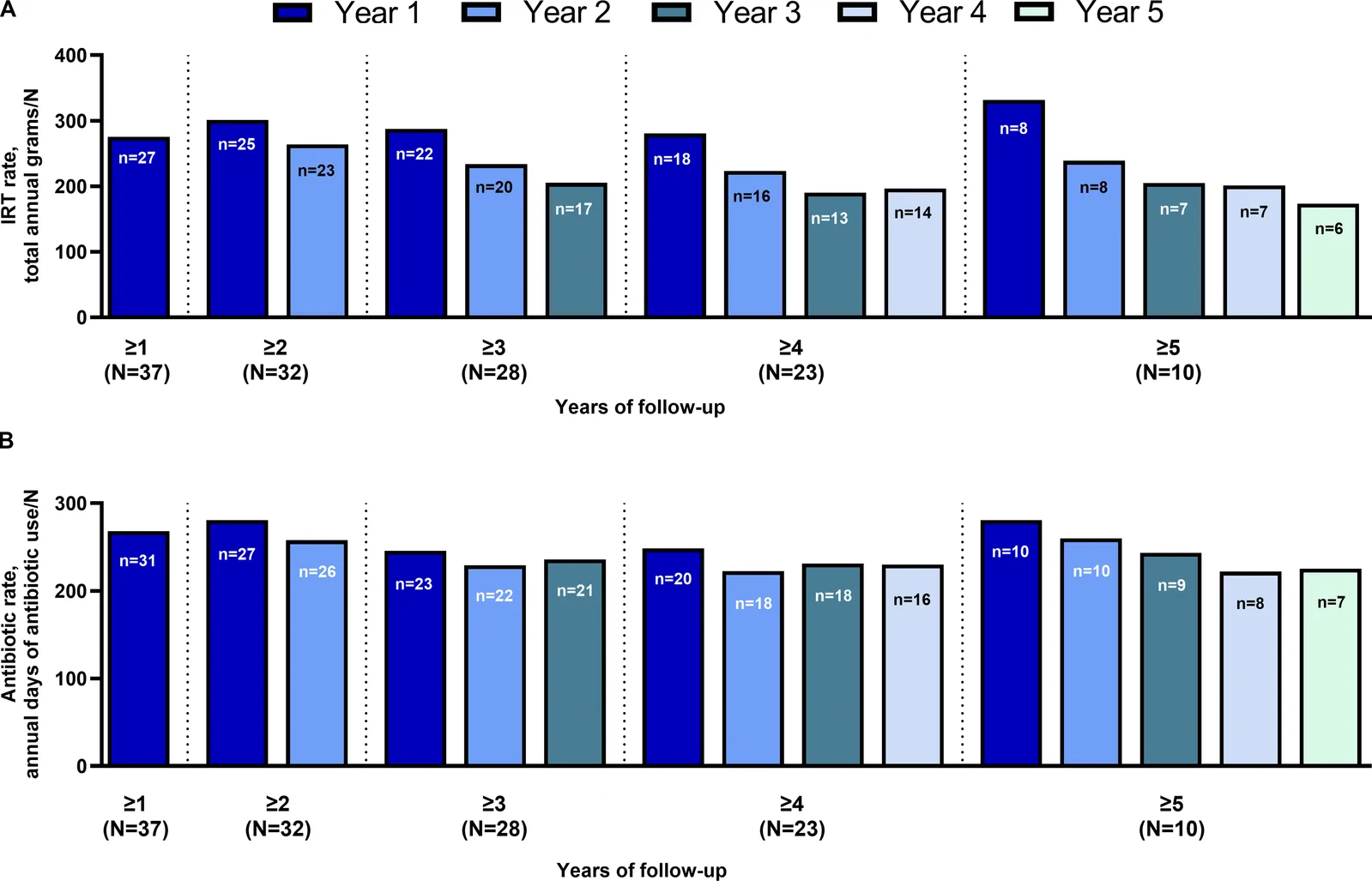
**Annual IRT and antibiotic use during treatment with leniolisib. (A and B)** Rate of (A) IRT or (B) antibiotic use by follow-up duration category within the OLE. *N*, number of patients in follow-up category; *n*, number of patients receiving IRT or antibiotic use.

27 patients (73%) received IRT at the start of the OLE study; of those, 10 patients (37%) discontinued and 2 patients achieved ≥25% reduction. No patients started IRT during the OLE. Patients who completed at least 1 year of follow-up experienced an 11% decrease in IRT consumption (in grams) with each year of leniolisib treatment (P = 0.0095). In patients with at least 5 years of follow-up (*n* = 10), the annual rate of IRT use decreased from 331.8 g at year 1 to 173.2 g by year 5, corresponding to a 48% decrease (Fig. 5 A). Days of antibiotic use remained stable, with a 2% decline per year of treatment (P = 0.1934). In patients with at least 5 years of follow-up (*n* = 10), the rate of annual antibiotic use decreased from 280.6 days at year 1 to 225.4 days at year 5 of treatment, corresponding to a 20% decrease (Fig. 5 B).

### Health-related QoL

**Figure 6.**
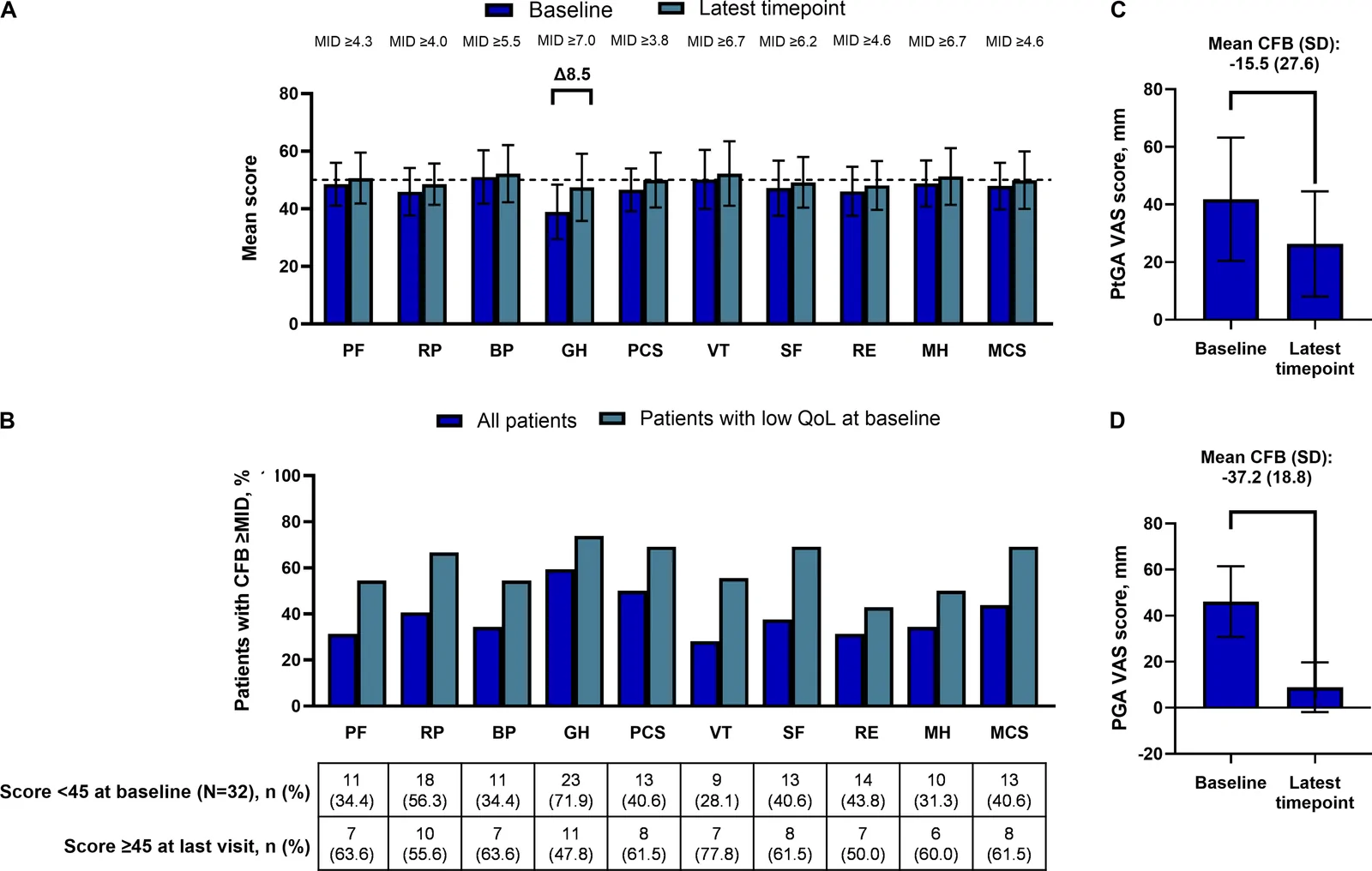
**Changes in QoL over time. (A)** Normed subscale and component scores from the SF-36 survey, with higher scores indicating greater health-related QoL. Differences in scores between baseline and the latest time point that meet or exceed MIDs are illustrated with a delta symbol (Δ) followed by the value of the difference. The dotted line indicates the general US population average. **(B)** All patients and patients with low SF-36 scores (<45) at baseline with clinically relevant changes in SF-36 scores at their last visit. The bottom table shows responder analysis of patients with low SF-36 scores at baseline. **(C and D)** (C) Mean patient and (D) physician global assessment scores are shown, with lower scores indicating a reduction in disease activity. *n* values in C and D are 37 and 32, respectively. Baseline was defined as the start of either the DFT or RCT. If a patient had a treatment gap >42 days between the DFT or RCT and the OLE, or they did not participate in either trial. ED 1 values were used as baseline. Error bars are SD. BP, bodily pain; CFB, change from baseline; GH, general health; MH, mental health; MID, minimally important difference; PF, physical functioning; PGA, physician global assessment; PtGA, patient global assessment; QoL, quality of life; RCT, randomized controlled trial; RE, role (emotional); RP, role (physical); SF, social functioning; VT, vitality.

Health-related QoL was assessed through patient- and physician-reported outcomes. The 36-Item Short Form Health Survey (SF-36) general health norm-based score increased by 8.5 points, which is considered a clinically meaningful improvement in patients with IEI (Fig. 6 A) (22, 23). At baseline, 23 out of 32 (72%) patients reported a low general health score. To examine changes in patients with low SF-36 scores at baseline, we performed a responder analysis that revealed a higher percentage of these patients experienced a minimally important difference in change from baseline in all categories of the SF-36 when compared with the entire cohort (Fig. 6 B). With the exception of general health, ≥50% of patients who had low QoL at baseline reached a score ≥45 by their last visit. The largest improvement occurred in the vitality category, where seven of the nine patients (78%) with a low vitality score at baseline reached a score of ≥45 by their last visit. Among patients with a below-normal physical component summary (PCS; *n* = 13) or mental component summary (MCS; *n* = 13) score at baseline, 62% achieved a score ≥45 by their last visit (*n* = 8 for each component summary score).

Overall disease activity as assessed by patient and physician global assessments showed reductions of 37% and 81%, respectively, by the latest time point with available data (Fig. 6, C and D).

### Immune profiling

**Figure 7.**
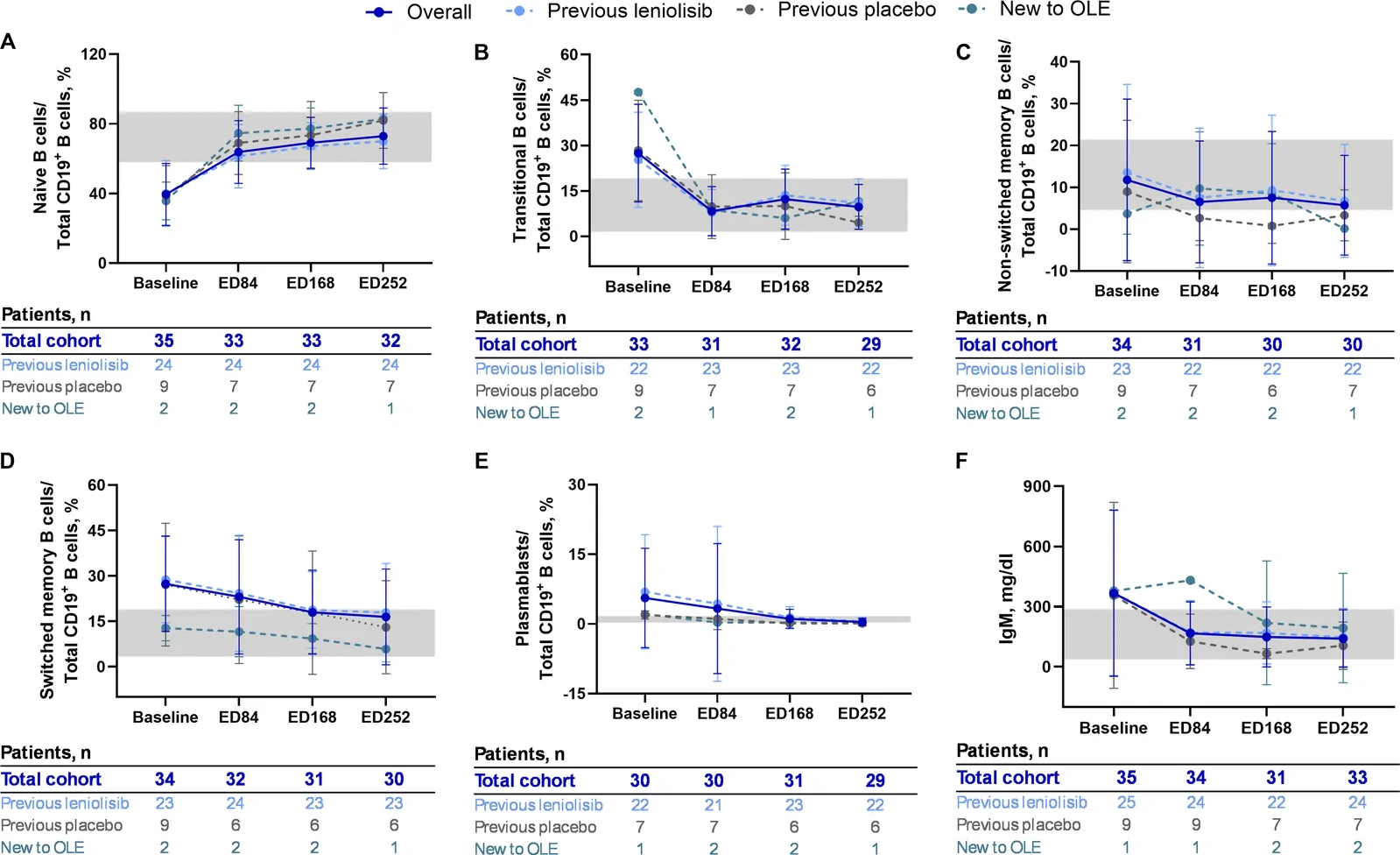
**Sustained changes in B cell subsets. (A)** Mean naïve B cell percentages (CD19^+^CD27^−^CD10^−^) over time. **(B)** Mean transitional B cell percentages (CD19^+^CD27^−^CD10^+^) over time. **(C)** Mean non-switched memory B cell percentages (CD19^+^CD27^+^IgD^+^) over time. **(D)** Mean switched memory B cell percentages (CD19^+^CD27^+^IgD^−^) over time. **(E)** Mean plasmablast percentages (CD19^+^CD27^+^CD38^++^) over time. **(F)** Mean IgM values over time. Patients are grouped into the following categories: overall (total cohort), previous leniolisib (received leniolisib prior to OLE), previous placebo (received placebo prior to OLE), and new to OLE (were not enrolled in prior leniolisib trials). Gray boxes indicate reference ranges, which were obtained from literature (panels A–E) or the clinical study report (panel F) (24). In F, for individual data that were reported as less than a certain value, the actual number was entered to graph the data. Error bars are SD.

Clinical and laboratory outcomes that can impact disease burden and QoL, such as pathognomonic B and T cell subsets, were also examined. Overall, changes were consistent with improvements reported in prior interim analyses (21). In the B cell compartment (Fig. 7, A–E), the mean percentage of naïve B cells, transitional B cells, memory B cells, and plasmablasts reached the reference range during the study and remained within range for the remainder of the study (24). Elevated IgM levels (Fig. 7 F) normalized and remained within the laboratory reference range throughout the remainder of the study.

To examine changes in patients with low naïve B cells (<48%) at baseline, we performed a responder analysis. Patients who had a ≥25% increase in naïve B cells compared with baseline were considered responders. 20 of 24 patients (83.3%) who had <48% naïve B cells at baseline met the responder threshold at the latest time point.

**Figure 8.**
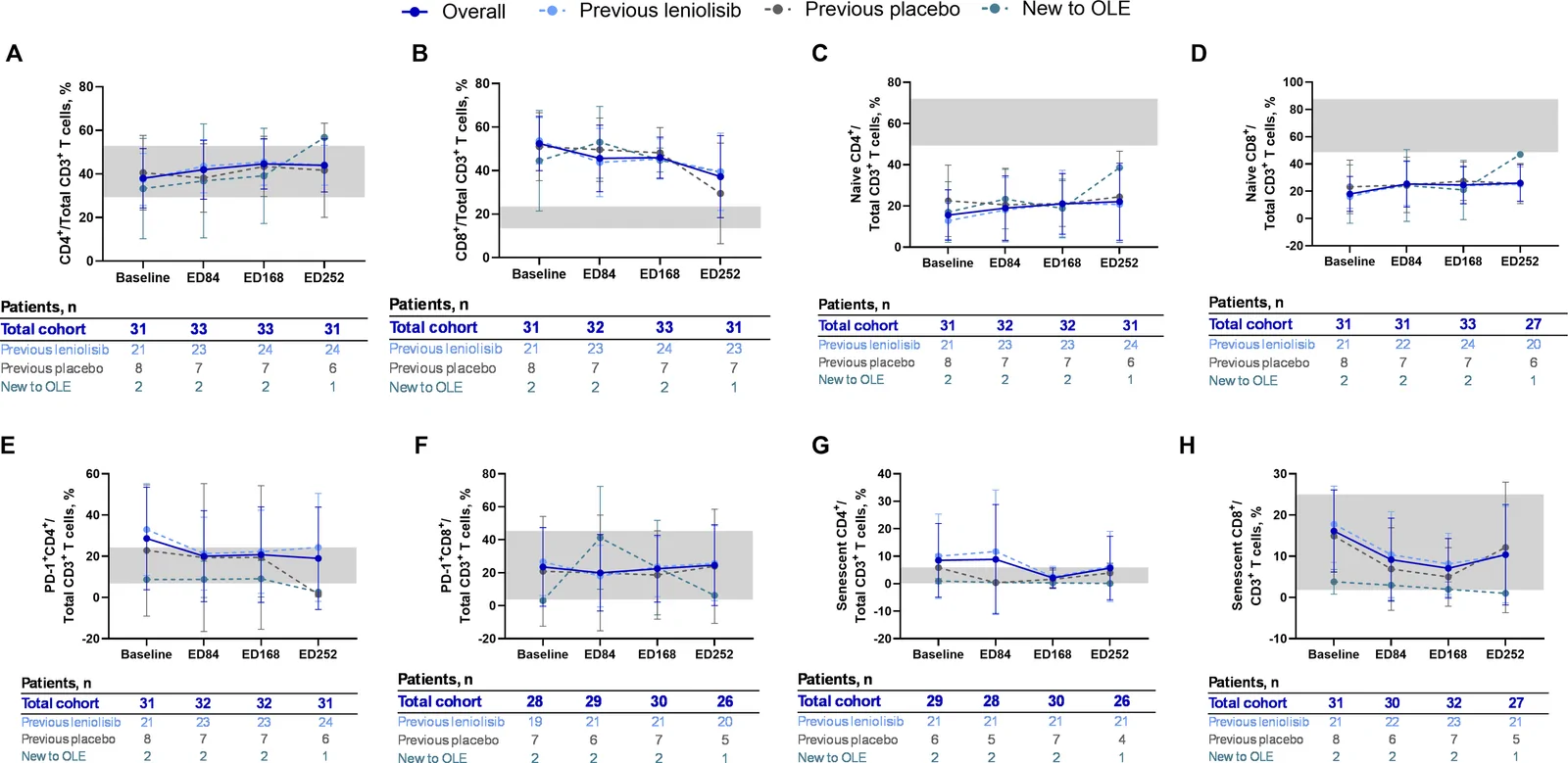
**Sustained changes in T cell subsets. (A and B)** (A) Mean CD4^+^ and (B) CD8^+^ T cell percentages over time. **(C and D)** (C) Mean naïve CD4^+^ and (D) naïve CD8^+^ T cell percentages (CD45RA^+^CD62L^+^) over time. **(E and F)** (E) Mean PD-1^+^CD4^+^ and (F) PD-1^+^CD8^+^ T cell percentages over time. **(G and H)** (G) Mean senescent (CD57^+^) CD4^+^ and (H) senescent CD8^+^ T cell percentages over time. Patients are grouped into the following categories: overall (total cohort), previous leniolisib (received leniolisib prior to OLE), previous placebo (received placebo prior to OLE), and new to OLE (were not enrolled in prior leniolisib trials). Gray boxes indicate reference ranges, which were obtained from either literature (panels A–F) or personal communication (panels G and H; Manish Butte).

In the T cell compartment (Fig. 8), mean percentages of CD4^+^ T cells, PD-1^+^CD8^+^ T cells, and CD8^+^CD57^+^ senescent T cells remained within the reference range throughout the study (25) (senescent T cells reference range from Manish Butte, personal communication). Mean percentage of CD8^+^ T cells slightly decreased during the study, while PD-1^+^CD4^+^ T cells and senescent CD4^+^ T cells reached reference range and remained within range for the remainder of the study (25). Naïve T cells remained below reference throughout the study (25).

### Lymphoproliferation

**Figure 9.**
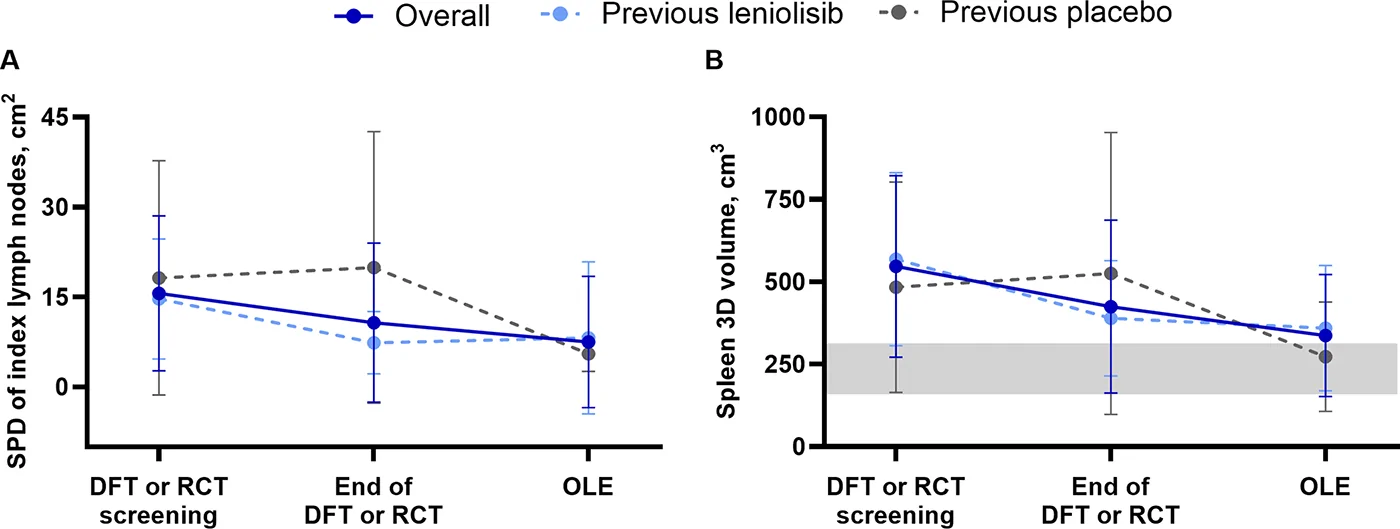
**Changes in lymphoproliferation parameters. (A)** Mean untransformed SPD of index lymph nodes. Reference range (≤1.5 × 1.5 cm) not shown, as up to six lymph nodes may be counted per patient. *n* values for total cohort (overall), prior leniolisib, and prior placebo are as follows: 30, 22, and 8. **(B)** Mean spleen 3D volume. Patients are grouped into the following categories: overall (total cohort), previous leniolisib (received leniolisib prior to OLE), and previous placebo (received placebo prior to OLE). The gray box in B indicates the reference range for adults. *n* values for total cohort (overall), prior leniolisib, and prior placebo are as follows: 31, 23, and 8. Error bars are SD. Imaging for the OLE study was optional and collected at either ED 168 or 252. Two patients who were not previously enrolled in the DFT or the RFT did not have available baseline images and were excluded from the analysis.

Consistent with prior interim analyses, reductions in lymphoproliferation were observed (21). Mean index lymph node sum of product of diameters (SPD; *n* = 30) decreased 50% for the total cohort compared with baseline, with mean index lymph node size decreasing by 8.13 cm^2^ (Fig. 9 A). Spleen 3D volume (*n* = 31) approached the reference range, reducing 36% compared with baseline (Fig. 9 B) (26).

To further examine changes in lymphoproliferation, we performed a responder analysis. Lymph node size reduction of either ≥30% (adults) or ≥45% (adolescents) at ED 168 or 252 was the benchmark for responders. 24 of 30 patients (80%) with enlarged lymph nodes at baseline met the responder threshold (data not shown). A reduction in spleen volume of either ≥27.5% (adult) or ≥35% (adolescent) at ED 168 or 252 was the benchmark for responders. 16 of 24 patients (67%) with an enlarged spleen at baseline met the responder threshold.

**Figure 10.**
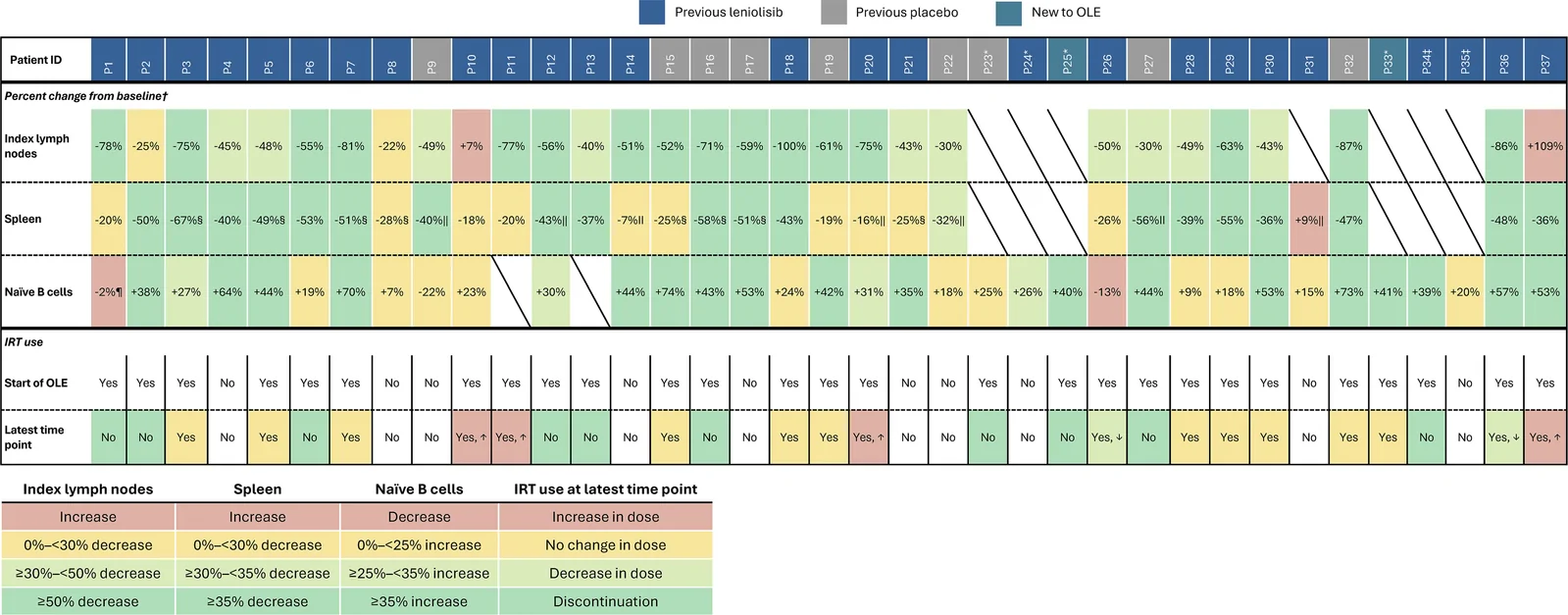
**Individual response to leniolisib treatment.** Slashed line indicates data were not available to determine percent change from baseline. ID, identification; P, patient; RCT, randomized placebo-controlled trial. *No imaging was completed in DFT or RCT. †OLE baseline was defined as the start of either the DFT or RCT. For naïve B cells, if a patient had a treatment gap >42 days between the DFT or RCT and the OLE, or they did not participate in either trial, ED 1 values were used as baseline. ‡No imaging completed in OLE. §Spleen volume normalized (<314.5 cm^3^) by OLE assessment in a patient with splenomegaly at baseline. ||Normal spleen volume (<314.5 cm^3^) at baseline and at OLE assessment. ¶ED 1 was used as baseline for this patient as they had a >42-day gap between studies, but the patient continued to receive leniolisib between the studies. Naïve B cells at this time point were high (92.5%).

Individual patient-level changes in lymphoproliferation and naïve B cells are illustrated in Fig. 10.

## Discussion

Results from this final report reinforce and expand upon outcomes reported from prior interim analyses of the OLE study. Overall, long-term administration of leniolisib was generally well tolerated, with durable improvements in lymphocyte subsets and lymphoproliferation noted. Results from responder analyses for naïve B cells and lymphoproliferation further strengthen clinically meaningful changes reported in such outcomes. Expanding upon the first interim analysis of the OLE, annual infection rate and IRT use significantly reduced with each year of leniolisib treatment. Notably, the positive, long-term impact of leniolisib on clinical manifestations was further bolstered by improvements in QoL. With up to 7 years of leniolisib exposure, this work represents the longest follow-up study assessing a PI3Kδ inhibitor in patients with APDS.

As leniolisib binds and inhibits the p110δ subunit of the PI3Kδ complex, it targets the underlying hyperactivity that is the hallmark of the disease (1, 6, 27). Prior studies have shown that targeting this hyperactivity with leniolisib elicits favorable outcomes in disease markers, including improvements in pathognomonic B and T cell subsets and lymphoproliferation (19, 20, 21). Improvements in clinical and laboratory outcomes may be due to the precise match of the mechanism of action of leniolisib to the mechanism of disease in APDS, which also likely contributes to the overall tolerability of leniolisib (27). Though AEs were experienced by nearly 92% of patients, most were grade 1 (87%) or 2 (68%), with only two patients discontinuing leniolisib due to AEs. Throughout the OLE, only five patients (14%) reported TRAEs, none of which were reported as serious. Overall, <30% of patients had grade 3 or greater AEs following exposure to leniolisib, further supporting its long-term tolerability. Tolerability is often a concern in patients with cancer who are treated with PI3Kδ inhibitors, where severe colitis or diarrhea AEs have been reported in 5–23% of patients (27). Over a span of up to 7 years, only one grade 3 colitis AE was reported in a patient with a complicated medical history that included ongoing gastrointestinal disease. We hypothesize that AEs commonly associated with PI3Kδ inhibitors in other populations may be minimized in patients with APDS, as their baseline hyperactive PI3Kδ is not completely inhibited but attenuated, with activity reaching physiologic range with leniolisib (27).

Another concern with PI3Kδ inhibition is genomic instability and the development of malignancy. However, an ex vivo study linking PI3Kδ inhibition with increased expression of activation-induced cytokine deaminase (AID) used cells that did not exhibit the hyperactive PI3Kδ signaling that is present in patients with APDS (28). PI3Kδ inhibition did not normalize but instead bluntly suppressed pathway activity in these cells. On the contrary, B cells expressing the APDS variant *PIK3CD* E1021K displayed decreased AID expression and chromosomal translocations, suggesting that PI3Kδ inhibition may not produce harmful levels of genomic instability in patients with APDS. While one patient treated with leniolisib in the OLE developed Hodgkin’s lymphoma at ED 750, an independent, multidisciplinary panel of experts concluded that it was unlikely that leniolisib contributed to the development of malignancy (29). The patient had several risk factors, including age, ethnicity, weight, history of EBV infections, family history, as well as leniolisib adherence concerns and a gap in treatment, that collectively suggested onset of lymphoma was unrelated to leniolisib use. Age and disease duration likely contributed to the development of suspected transitional cell carcinoma in another patient in the OLE. At 55 years of age, he enrolled in the OLE as the oldest patient to receive leniolisib treatment after living with APDS for ∼19 years. The risk for development of lymphoma as well as other progressive manifestations associated with APDS that are discussed later emphasizes the need for long-term registry data that retrospectively compares patients who were and were not treated with leniolisib, particularly those with lymphoproliferation, to elucidate potential beneficial effects for preventing cancer development.

The occurrence of Hodgkin’s lymphoma and suspected transitional cell carcinoma in one patient each during the OLE are noteworthy, as an accelerated decline in survival has been associated with concurrent lymphoma in patients with APDS (30). To better understand the association between advanced disease and adverse outcomes, the European Society for Immunodeficiencies (ESID) defined prognostic factors for severe disease course in APDS as severe invasive infections, immune dysregulation, chronic lung disease, or malignancy. The risk for development of severe disease increases with age and years since first clinical disease manifestation (8). Similar findings are reported in patients with complicated common variable immunodeficiency (CVIDc) who have higher mortality rates than those with an infection-only phenotype. Specifically, mortality in patients with CVIDc is associated with lymphoma, any form of hepatitis, lung impairment, and gastrointestinal disease (31, 32). Collectively, these data suggest certain manifestations along with disease duration may be associated with a high risk for development of adverse outcomes, but further studies are needed to examine and define such prognostic factors for patients with APDS.

Similar to prior interim analyses, final results from the OLE further support sustained disease modification spanning from pathognomonic B and T cells to clinical manifestations (21). While changes in lymphocyte subsets and lymphoproliferation are crucial, other aspects of disease burden such as infections and IRT use were also assessed. Here, we showed a significant reduction in the annualized rate of infections as well as respiratory infections with each additional year of leniolisib. Leniolisib also significantly reduced annual infection rates in a study comparing patients in the OLE to an external cohort enrolled in the ESID registry (33). In the current study, this decrease was observed in conjunction with a significant reduction in IRT, with two patients reducing their dose by ≥25% and approximately a third of patients discontinuing use. The annual rate of antibiotic use also decreased, but it was not statistically significant. This lack of significance could, in part, be explained by the fact that patients with bronchiectasis (46% in the OLE) likely had limited changes in antibiotic use, as the European Respiratory Society guidelines recommend these patients be treated with prophylactic antibiotics (34). The decrease in annual respiratory infections is noteworthy, as nearly all patients with APDS experience recurrent sinopulmonary infections (8). Moreover, patients with APDS have reported several restrictions due to infections, including social and leisure activities, and have concerns surrounding frequent IRT administration (13). Recurrent infections also carry a financial burden, costing a patient with APDS an average of $31,711 per year according to 2023 pricing (35). Therefore, a decrease in overall infections and IRT use with each year of leniolisib treatment likely reflects the positive changes in QoL that were observed during the study.

Patients with IEIs such as APDS have a high disease burden and report a lower perception of general health compared with healthy individuals, highlighting the importance of determining the impact of leniolisib on QoL (13, 36). Overall, 62% of patients with low PCS or MCS scores (<45) at baseline reached a score of ≥45 by their last visit. This positive change in SF-36 scores is meaningful as it indicates the majority of patients with low QoL at baseline reached levels observed in the general population during treatment with leniolisib.

While we report durable improvements in disease markers of APDS in patients receiving leniolisib, gaps in knowledge remain. Infection rates decreased with each year of leniolisib treatment, but the extent of patient-level variability remains unclear and warrants within-subject analyses to further support long-term durability of treatment response. Additionally, interpretation of long-term outcomes is limited by the absence of follow-up data for patients who discontinued the OLE and continued use of leniolisib outside of the trial.

Over the course of the OLE, 2 out of 37 patients (5%) developed malignancy over a mean of 4.1 years of follow-up, while historical data from the ESID APDS registry reports 6 of 80 patients with a follow-up visit (8%) developed malignancy over a mean of 2.6 years. Studies assessing the rate of malignancy in patients treated with leniolisib compared with those not treated or treated with other therapies such as mTOR inhibitors are warranted. In addition to malignancy, the impact of leniolisib on other progressive manifestations such as end-organ damage remains unclear. Aside from the one patient experiencing exacerbation of bronchiectasis throughout the OLE and the three patients from the original DFT whose bronchiectasis remained stable out to year 6 of treatment, studies involving larger cohorts of patients have yet to be completed (37). However, future ESID registry updates along with a recently constructed APDS registry (APDS-CHOIR) could shed light on the rate of malignancy as well as other progressive manifestations amongst this patient population, including those receiving leniolisib. In addition to the impact of leniolisib on progressive manifestations, studies examining use of the PI3Kδ inhibitor for treatment of pediatric patients <12 years with APDS are crucial, as early onset can be a risk factor for developing severe disease. Currently, two pediatric trials are assessing the safety and efficacy of leniolisib in patients with APDS between the ages of 1–6 and 4–11 years (NCT05693129 and NCT05438407, respectively) (7).

Within 10 years of the characterization of APDS, leniolisib was developed as a novel, targeted treatment option (1, 38). Improvements in immune subsets and lymphadenopathy in patients with APDS who received leniolisib were illustrated across multiple clinical trials including a DFT, RCT, and OLE study (19, 20, 21). In this final report from the OLE, we observed positive and sustained changes in health-related QoL and clinical outcomes, with no additional unexpected safety concerns, supporting the use of leniolisib as a long-term disease-modifying treatment for APDS.

## Materials and methods

### Patients and treatment

Patients with APDS aged ≥12 years who had completed the DFT or the RCT (NCT02435173) or participated in other PI3Kδ inhibitor studies and met the eligibility criteria for the leniolisib trials were enrolled in the OLE (NCT02859727) from September 2016 to August 2021 and received 70 mg of leniolisib twice daily. Male and female patients aged 12–75 years old, weighing ≥45 kg, and who had a pathogenic variant in *PIK3CD* or *PIK3R1* along with clinical manifestations of APDS were eligible for the study. Previous or concurrent use of a mTOR inhibitor was allowed >7 wk before the first dose, while short-term use (≤5 days) of an mTOR inhibitor was allowed up to 1 mo before enrollment. Glucocorticoids equivalent to ≤25 mg prednisone per day were allowed up to 2 wk before the first dose and throughout the study. Patients were also excluded if they withdrew consent from either the DFT or RCT, were pregnant or lactating, or had a history of hypersensitivity to study treatment, electrocardiogram abnormalities, or malignancy of any organ system. The exception to the latter was three of the original six patients from the DFT who had a history of lymphoma that resolved prior to entering the study. Patients who did not participate in the RCT but were previously treated with a PI3Kδ inhibitor other than leniolisib were excluded if vital signs were not within normal limits or body weight was <45 kg at screening. Additionally, a patient was excluded if any of the following infections were present at screening: tuberculosis, hepatitis B, hepatitis C, or human immunodeficiency virus.

Six patients were enrolled from the DFT. 29 were enrolled from the RCT; 20 were previously exposed to leniolisib, and 9 received placebo. Two patients were newly enrolled into the OLE study and had historical PI3Kδ inhibitor exposure that was not leniolisib.

### Trial design

Patients from the US, Europe, and Russia were enrolled in a long-term, single-arm, OLE study. The latest visit for any patient within the final OLE study was January 30, 2025.

### End points and assessments

The primary endpoint of the OLE study was to evaluate the long-term safety and tolerability of leniolisib, which included AEs that were assessed at every scheduled visit throughout the entire study. Health-related QoL was a secondary endpoint assessed via the SF-36 and patient and physician global assessments. QoL assessments were completed at every scheduled visit throughout the entire study. Additional secondary endpoints included frequency of infections and annual IRT and antibiotic use, which were reported by trial investigators as AEs or concomitant medications, respectively. Antibiotic use included both prophylactic and acute use. Reduction of IRT and antibiotic use was determined by the trial investigator based on perceived progress of the patient.

Exploratory outcomes included reduction of lymphoproliferation that was assessed via changes in spleen volume and lymph node size, as determined through magnetic resonance imaging (MRI) or computed tomography (CT), and B and T cell immunophenotyping. Immunophenotyping was completed through ED 252 or 364. Imaging used to determine lymphoproliferation was completed at either ED 168 or ED 252, and images were obtained only for patients previously enrolled in either the DFT or the RCT. Six patients were excluded from lymphoproliferation analyses; four patients (two were not enrolled in the DFT or the RCT, and another two did not have informed consent during the RCT) did not have available baseline images, while two patients did not have available OLE images.

### Data analysis

Except for infections, IRT, and antibiotic rates, assessments were descriptive in nature, as the OLE was a single-arm study and results are reported as readouts over time. OLE baseline was defined as the start of either the DFT or RCT. For immunophenotyping and the naïve B cell responder analysis, if a patient had a treatment gap >42 days between the DFT or RCT and the OLE, or they did not participate in either trial, ED 1 values were used as baseline. Annual rates of infection, IRT use, and antibiotic use were assessed using one-sided log-linear negative binomial models that included an effect for time at the start of infections (in years) and an offset for the amount of follow-up time available within each year for each patient. Data were included for each patient up to their final partial year. A repeated statement with an exchangeable correlation structure was used to model the intra-patient correlation. If a start day was missing, it was imputed as the start of the month. If no infections or relevant medications were experienced in a year, the count was set to 0 for that year. MRI or CT scans, as well as immune subset data, were further stratified by previous exposure to leniolisib or placebo.

Responder analyses for naïve B cells, index lesion SPD, and spleen 3D volume were performed to examine changes in lymphoproliferation. An e-Delphi panel of 24 APDS experts identified treatment outcome changes indicating meaningful patient benefit over five rounds of online surveys (39). Panelists identified within-patient clinically meaningful improvements in adult (≥18 years) and adolescent (12 to <18 years) patients. Change from baseline was measured to discern responders from nonresponders. Both adults and adolescents with a ≥25% increase in naïve B cells were considered responders. Adult patients with a ≥30% reduction in lymph node size and ≥27.5% in spleen volume were considered responders. Adolescent patients with a ≥45% reduction in lymph node size and ≥35% in spleen volume were considered responders. An enlarged spleen was defined as spleen volume >314 ml at baseline.

Changes in QoL were assessed by examining differences in SF-36 normed subscale and component scores between baseline and the latest time point. Scores were considered meaningful if they met or exceeded minimally important differences that were defined as follows: PCS, 3.8; MCS, 4.6; physical functioning, 4.3; role (physical), 4.0; bodily pain, 5.5; general health, 7.0; vitality, 6.7; social functioning, 6.2; role (emotional), 4.6; and mental health, 6.7 (22, 23). A responder analysis of SF-36 norm-based, or component summary scores was also conducted using patients with a score of <45 at baseline. A patient fulfilled responder criteria if they reached a norm-based or component summary score of ≥45 by their last visit.

All graphs were plotted using GraphPad Prism v10.2.3. A P value <0.05 was considered significant.

### Trial oversight

Novartis Pharmaceuticals Corporation, in collaboration with investigators, designed the study. Novartis oversaw trial conduct and analyzed data, with Pharming Group N.V. acquiring rights to leniolisib in 2019 and full trial management in December 2022. The study was conducted and reported in accordance with the protocols (including the statistical analysis plan). Data for the analysis were gathered locally, and protocol-defined laboratory samples and imaging were processed centrally. The trial was conducted in accordance with the Declaration of Helsinki and the International Council for Harmonization Good Clinical Practice guidelines. Independent ethics committees or institutional review boards at each center approved the protocol. Patients and/or their guardians provided written informed consent/ascent. An independent monitoring committee monitored safety, data integrity, and protocol compliance. The study population is representative (e.g., sex, age, race, and ethnicity) of the population of patients with APDS at large.

### Online supplemental material

Table S1 summarizes patient characteristics and demographics at baseline of the OLE. Table S2 lists the most common AEs in ≥2 patients. Table S3 reports serious AEs and TRAEs reported during the OLE.

## Supplementary Material

Table S1 shows OLE baseline characteristics and demographics.

Table S2 shows the most common AEs in ≥2 patients.

Table S3 shows serious AEs and TRAEs.

## Data Availability

The data are available upon reasonable request. Researchers who have a methodologically sound research proposal should send inquiries or requests to info@pharming.com. Data requestors may be required to sign a data access agreement.
