# Supplementary material for: Open-label extension study of leniolisib in activated PI3Kδ syndrome: Long-term efficacy and safety
Source: J Hum Immun. 2026 Oct 14;3(1):e20260040. doi: 10.70962/jhi.20260040 (PMC13664708; doi:10.70962/jhi.20260040)
Supplement: Supplementary file 1 [file jhi_20260040_tables1.docx]

**Table S1. OLE baseline characteristics and demographics**

| Patient Characteristic | Value |
| --- | --- |
| **Previous study participation, n (%)** | |
| Dose-finding trial | 6 (16.2) |
| Randomized controlled trial | 29 (78.4) |
| *Placebo* | 9 (24.3) |
| *Leniolisib* | 20 (54.1) |
| Other PI3Kδ inhibitor studies | 2 (5.4) |
| **Age at time of study, median (range), y** | 20.0 (12-55) |
| <18 years, n (%) | 12 (32.4) |
| **Male;female, n** | 21:16 |
| **APDS1:APDS2, n** | 30:7 |
| **Time since APDS diagnosis, median (range), y^a^** | 2.0 (0.2-6.4) |
| **Medical history of interest, n (%)** | |
| Infections | 32 (86.5) |
| Lymphoproliferation^b^ | 25 (67.6) |
| *Lymphadenopathy* | 24 (64.9) |
| *Splenomegaly* | 15 (40.5) |
| *Lymphoid hyperplasia* | 2 (5.4) |
| Cytopenia^c^ | 20 (54.1) |
| Chronic gastrointestinal disease^d^ | 19 (51.4) |
| Chronic pulmonary disease^e^ | 21 (56.8) |
| *Bronchiectasis* | 17 (46.0) |
| *Asthma* | 10 (27.0) |
| Fatigue | 12 (32.4) |
| **Medications of interest ongoing at OLE baseline, n (%)** | |
| Immunoglobulin replacement therapy | 27 (73.0) |
| Antibiotics | 22 (59.5) |
| Glucocorticoids^f^ | 21 (56.8) |
| *Oral* | 7 (18.9) |
| *Inhaled/intranasal* | 14 (37.8) |
| *Topical* | 6 (16.2) |
| **Prior sirolimus use, n (%)^g^** | 8 (21.6) |

APDS, activated PI3Kδ syndrome; OLE, open-label extension; SD, standard deviation. ^a^Missing information for 1 patient. ^b^Lymphoproliferation included lymphadenopathy, lymphoproliferative disease, splenomegaly, hepatomegaly, hepatosplenomegaly, lymphoid hyperplasia, lymphocytic infiltration, adenoidectomy, tonsillectomy, adenotonsillectomy and adenoidal hypertrophy. ^c^Cytopenias included anemia, lymphopenia, neutropenia or thrombocytopenia. ^d^Chronic gastrointestinal disease included celiac disease, enteritis, gastritis, duodenitis, colitis, gastroesophageal reflux disease, eosinophilic esophagitis, chronic/recurrent nausea, vomiting or diarrhea, failure to thrive, recurrent GI pain, recurrent constipation and recurrent hematochezia. ^e^Chronic pulmonary disease included asthma, bronchiectasis, interstitial lung disease, pulmonary fibrosis, restrictive pulmonary disease and small airways disease. ^f^Glucocorticoid doses equivalent to ≤25 mg per day of prednisone were allowed within 2 weeks before the first dosing and throughout the study. ^g^Previous or concurrent use of an mTOR inhibitor was allowed >7 weeks before first dose while short-term use (≤5 days) of an mTOR inhibitor was allowed up to 1 month before enrollment.
