# Supplementary material for: Open-label extension study of leniolisib in activated PI3Kδ syndrome: Long-term efficacy and safety
Source: J Hum Immun. 2026 Oct 14;3(1):e20260040. doi: 10.70962/jhi.20260040 (PMC13664708; doi:10.70962/jhi.20260040)
Supplement: Supplementary file 2 [file jhi_20260040_tables2.docx]

**Table S2. Most common adverse events in ≥2 patients**

| Preferred Term | Incidence of AE, n (%) | | | Preferred Term | Incidence of AE, n (%) | | | Preferred Term | Incidence of AE, n (%) | | |
| --- | --- | --- | --- | --- | --- | --- | --- | --- | --- | --- | --- |
|  | **Any** | **G1–2** | **G≥3** |  | **Any** | **G1–2** | **G≥3** |  | **Any** | **G1–2** | **G≥3** |
| COVID-19 | 12 (32.4) | 11 (29.7) | 1 (2.7) | Pharyngitis | 4 (10.8) | 4 (10.8) | – | Flank pain | 2 (5.4) | 1 (2.7) | 1 (2.7) |
| Upper RTI | 10 (27.0) | 10 (27.0) | – | Pneumonia | 4 (10.8) | 3 (8.1) | 1 (2.7) | Folliculitis | 2 (5.4) | 2 (5.4) | – |
| Pyrexia | 9 (24.3) | 8 (21.6) | 1 (2.7) | Urinary tract infection | 4 (10.8) | 3 (8.1) | 1 (2.7) | Hematochezia | 2 (5.4) | 1 (2.7) | 1 (2.7) |
| Headache | 8 (21.6) | 8 (21.6) | – | ALT increased | 3 (8.1) | – | 3 (8.1) | Herpes simplex | 2 (5.4) | 2 (5.4) | – |
| Otitis externa | 7 (18.9) | 7 (18.9) | – | Herpes Zoster | 3 (8.1) | 3 (8.1) | – | Hypokalemia | 2 (5.4) | 1 (2.7) | 1 (2.7) |
| Nasopharyngitis | 6 (16.2) | 6 (16.2) | – | Migraine | 3 (8.1) | 3 (8.1) | – | Influenza | 2 (5.4) | 2 (5.4) | – |
| Sinusitis | 6 (16.2) | 5 (13.5) | 1 (2.7) | Otitis media | 3 (8.1) | 3 (8.1) | – | Influenza-like illness | 2 (5.4) | 2 (5.4) | – |
| Abdominal pain | 5 (13.5) | 3 (8.1) | 2 (5.4) | Rash | 3 (8.1) | 3 (8.1) | – | Illusion | 2 (5.4) | 2 (5.4) | – |
| Arthralgia | 5 (13.5) | 5 (13.5) | – | Seasonal allergy | 3 (8.1) | 3 (8.1) | – | Joint injury | 2 (5.4) | 2 (5.4) | – |
| Rhinitis | 5 (13.5) | 5 (13.5) | – | Seborrheic dermatitis | 3 (8.1) | 3 (8.1) | – | Keratosis pilaris | 2 (5.4) | 2 (5.4) | – |
| RTI | 5 (13.5) | 5 (13.5) | – | Abdominal pain, upper | 2 (5.4) | 2 (5.4) | – | Ligament sprain | 2 (5.4) | 2 (5.4) | – |
| Vomiting | 5 (13.5) | 4 (10.8) | 1 (2.7) | Acute otitis media | 2 (5.4) | 2 (5.4) | – | Limb injury | 2 (5.4) | 2 (5.4) | – |
| Weight increased | 5 (13.5) | 2 (5.4) | 3 (8.1) | Allergic conjunctivitis | 2 (5.4) | 2 (5.4) | – | Lyme disease | 2 (5.4) | 2 (5.4) | – |
| Asthma | 4 (10.8) | 3 (8.1) | 1 | Alopecia | 2 (5.4) | 2 (5.4) | – | Lymphadeno-pathy | 2 (5.4) | 1 (2.7) | 1 (2.7) |
| Bronchitis | 4 (10.8) | 4 (10.8) | – | Anal fissure | 2 (5.4) | 1 (2.7) | 1 (2.7) | Myalgia | 2 (5.4) | 2 (5.4) | – |
| Cough | 4 (10.8) | 4 (10.8) | – | Anemia | 2 (5.4) | 1 (2.7) | 1 (2.7) | Nausea | 2 (5.4) | 2 (5.4) | – |
| Dental caries | 4 (10.8) | 4 (10.8) | – | AST increased | 2 (5.4) | – | 2 (5.4) | Non-cardiac chest pain | 2 (5.4) | 2 (5.4) | – |
| Diarrhea | 4 (10.8) | 4 (10.8) | – | Back pain | 2 (5.4) | 2 (5.4) | – | Obesity | 2 (5.4) | – | 2 (5.4) |
| Gastroenteritis | 4 (10.8) | 4 (10.8) | – | Conjunctivitis | 2 (5.4) | 2 (5.4) | – | Paresthesia | 2 (5.4) | 2 (5.4) | – |
| GERD | 4 (10.8) | 4 (10.8) | – | Dysphonia | 2 (5.4) | 2 (5.4) | – | Paronychia | 2 (5.4) | 2 (5.4) | – |
| Oral herpes | 4 (10.8) | 4 (10.8) | – | Dyspnea | 2 (5.4) | 2 (5.4) | – | Productive cough | 2 (5.4) | 2 (5.4) | – |
| Oropharyngeal pain | 4 (10.8) | 4 (10.8) | – | Epistaxis | 2 (5.4) | 2 (5.4) | – | Skin papilloma | 2 (5.4) | 2 (5.4) | – |

AE, adverse event; ALT, alanine aminotransferase; AST, aspartate aminotransferase; COVID-19, coronavirus disease 2019; G, grade; GERD, gastroesophageal reflux disease; RTI, respiratory tract infection.
