# Supplementary material for: Open-label extension study of leniolisib in activated PI3Kδ syndrome: Long-term efficacy and safety
Source: J Hum Immun. 2026 Oct 14;3(1):e20260040. doi: 10.70962/jhi.20260040 (PMC13664708; doi:10.70962/jhi.20260040)
Supplement: Supplementary file 3 [file jhi_20260040_tables3.docx]

**Table S3. Serious and treatment-related adverse events.**

| Preferred Term | Incidence of AE, n (%) |
| --- | --- |
| Serious adverse event^a^ | |
| Abdominal pain | 2 (5.4) |
| Pneumonia | 2 (5.4) |
| Acute pancreatitis | 1 (2.7) |
| Acute sinusitis | 1 (2.7) |
| ALT increased | 1 (2.7) |
| Anemia | 1 (2.7) |
| Anal fissure | 1 (2.7) |
| Anaphylactic reaction | 1 (2.7) |
| Angina | 1 (2.7) |
| Aspiration | 1 (2.7) |
| Asthma | 1 (2.7) |
| COVID-19 | 1 (2.7) |
| Cardiac arrest | 1 (2.7) |
| Colitis | 1 (2.7) |
| Deep vein thrombosis | 1 (2.7) |
| Dehydration | 1 (2.7) |
| Facial pain | 1 (2.7) |
| Febrile neutropenia | 1 (2.7) |
| Flank pain | 1 (2.7) |
| Gastroenteritis | 1 (2.7) |
| Hodgkin’s disease | 1 (2.7) |
| Hypocalcemia | 1 (2.7) |
| Hypotension | 1 (2.7) |
| Leukopenia | 1 (2.7) |
| Lymphadenopathy | 1 (2.7) |
| Muscular weakness | 1 (2.7) |
| Orthostatic hypotension | 1 (2.7) |
| Paresthesia | 1 (2.7) |
| Parotitis | 1 (2.7) |
| Periorbital cellulitis | 1 (2.7) |
| Pyrexia | 1 (2.7) |
| Reactive arthritis | 1 (2.7) |
| Respiratory disorder | 1 (2.7) |
| Sinusitis | 1 (2.7) |
| Soft tissue abscess | 1 (2.7) |
| Staphylococcal bacteremia | 1 (2.7) |
| Suicide ideation | 1 (2.7) |
| Transitional cell carcinoma | 1 (2.7) |
| Urinary tract infection | 1 (2.7) |
| Viral infection | 1 (2.7) |
| Vomiting | 1 (2.7) |
| Treatment-related adverse event^b^ | |
| Weight increased | 3 (8.1) |
| Arthralgia | 1 (2.7) |
| Hyperglycemia | 1 (2.7) |
| Neutrophil count decreased | 1 (2.7) |

^a^Six out of 10 patients reported multiple serious adverse events. ^b^One out of 5 patients experienced multiple treatment-related adverse events.

COVID-19, coronavirus disease 2019
